# From vector to allergen: exploring the immunology of tick-triggered α-Gal syndrome

**DOI:** 10.3389/fimmu.2026.1838920

**Published:** 2026-05-20

**Authors:** Julie Petry, Kyra Swiontek, Christiane Hilger

**Affiliations:** Department of Infection and Immunity, Luxembourg Institute of Health, Esch-sur-Alzette, Luxembourg

**Keywords:** red meat allergy, sensitization mechanism, tick immunity, tick-borne disease, α-Gal IgE, α-Gal syndrome

## Abstract

The α-Gal syndrome (AGS) is an emerging form of food allergy characterized by delayed hypersensitivity reactions to mammalian meat products and mediated by IgE antibodies specific to the carbohydrate galactose-α-1,3-galactose (α-Gal). Although α-Gal–specific IgG, IgM and IgA antibodies are generally present in humans as a consequence of continuous exposure to commensal microbiota and dietary sources, IgE sensitization to α-Gal occurs only in a subset of individuals. Epidemiological and experimental evidence has firmly linked this sensitization to tick bites. Multiple ticks across continents have been implicated in α-Gal sensitization, and α-Gal has been detected in tick midgut, hemolymph, and salivary glands; yet the precise origin of α-Gal within ticks and the immunological mechanisms that drive α-Gal-specific IgE production remain incompletely understood. In particular, it remains unclear how cutaneous exposure to ticks promotes IgE class switching against α-Gal, whereas lifelong gastrointestinal exposure to the same epitope does not elicit allergic sensitization. Despite a growing number of reviews addressing the clinical and epidemiological aspects of AGS, significant gaps persist in our understanding of the molecular and immunological pathways underlying disease development. This review aims to address these gaps by focusing specifically on the molecular and immunological pathways involved in α-Gal sensitization following tick bites, with particular emphasis on the innate and adaptive immune responses that drive the production of α-Gal-specific IgE. By integrating data from human studies, animal models and *in vitro* systems, a more cohesive understanding of the immune dynamics contributing to Th2-biased immune responses and sensitization begins to emerge. Ultimately, a detailed understanding of how the cutaneous environment, tick saliva components, and host factors synergize to induce a Th2-biased IgE sensitization is key to identifying diagnostic biomarkers and prevention approaches.

## 1 Introduction

The α-Gal syndrome (AGS) recently came into the spotlight upon the release of two reports by the Centers for Disease Control and Prevention (CDC) in 2023, qualifying AGS as ‘an important emerging public health problem’ and revealing limited knowledge of AGS among US health care providers, leading to underdiagnosis and inadequate patient care (1, 2).

The general lack of awareness of AGS among health care providers worldwide is understandable, given the atypical features of this allergic disease and its origins. Although AGS could be simply described as a red meat allergy, several factors make the picture actually more complex. The most atypical feature might be the delayed onset of allergic reactions after consumption of red meat by AGS patients. Unlike other food allergies, the allergic reactions in AGS are generally delayed from 2 to 6 hours, but in some cases up to 8 hours, or sometimes less than 2 hours depending on the type or quantity of ingested meat. For some patients, allergic reactions are not systematic after red meat consumption and are dependent on co-factors such as physical activity following the meal and/or consumption of alcohol or nonsteroidal anti-inflammatory drugs (NSAIDs) (3).

Furthermore, in general, peptide epitopes are the targets of specific IgE antibodies in food allergies, whereas in AGS specific IgE antibodies are directed against a single epitope consisting of a disaccharide: two galactose molecules linked by an α-1,3 bond, commonly called alpha-Gal (α-Gal). Generally, IgE reactivity against sugars or cross-reactive carbohydrate determinants (CCDs) does not have clinical relevance (4). The cross-linking of anti-α-Gal IgE on mast cells and basophils, however, leads to clinical manifestations in patients, ranging from mild to severe and including symptoms such as urticaria, angioedema, gastrointestinal and respiratory symptoms, and even anaphylaxis. The type and severity of symptoms is not only variable from one patient to another, but also from one episode to the next in the same individual (5). The first documented case of fatal anaphylaxis in an AGS patient impressively shows that isolated symptoms such as abdominal pain may not be recognized as AGS and that there is a clear need for more awareness among health care providers and the public (6).

Finally, AGS is the only allergy known to be initiated by a vector but not directed against the vector itself. It has been established that (i) sensitization occurs through tick bites, (ii) different tick species are capable of transmitting the α-Gal epitope in various regions of the globe, (iii) some tick species do not carry the α-Gal epitope and do not play a role in sensitization, (iv) after repeated tick bites, specific anti-α-Gal IgE antibodies rise in the serum of sensitized individuals and fall again after avoidance of tick bites (7). Other human parasites have been described to contain α-Gal, such as *Plasmodium* spp. sporozoites (8), *Leishmania* spp. (9), and *Trypanosoma cruzi* (10). Recently, the presence of the α-Gal epitope was also confirmed in the parasitic nematode *Anisakis* sp. (11), but the role of these parasites in α-Gal sensitization still needs to be demonstrated.

The scope of this review is to discuss the role of ticks in the etiology of AGS by compiling scientific knowledge obtained in human and animal research, to point to current knowledge gaps and offer guidance for future research. While the main focus is drawn upon immunological shifts happening after tick bites, diving deep into immunological interplays of humoral and cellular actors of the immune response in animal and human models, the current state of knowledge of AGS epidemiology is also highlighted.

## 2 Discovery and key milestones

As early as the 1930s, Karl Landsteiner, Nobel Prize winner in Physiology or Medicine for the discovery of ABO blood groups, described a B-like antigen expressed on mammalian cells, except for humans, apes and Old World monkeys (12). More than thirty years later, the presence of the non-reducing terminal sequence Galα1-3Galβ1-4GlcNAc-R, subsequently known as the α-Gal epitope, was reported on a glycolipid from rabbit red blood cells (13). The structures of rabbit glycolipids were further characterized in the following two decades and the α-Gal epitope was also detected on bovine, pig and sheep glycolipids (14). Since the 1980s, several research groups have detected the presence of the α-Gal epitope on mammalian proteins by glycomics analysis: thyroglobulin from several species (15, 16), as well as mouse and bovine laminin (17, 18).

In 1984, Uri Galili and his colleagues published the first articles describing a natural polyclonal anti-α-Gal IgG antibody purified from healthy human serum (19). This polyclonal antibody was used to detect α-Gal on thyroglobulin, fibrinogen and immunoglobulin G from several mammalian species (20), porcine β1 integrin subunit (21), but also on several species of microbiota (22). Since the discovery of the α-Gal epitope and the natural anti-Gal response in humans, it became quickly clear that α-Gal was an important barrier to xenotransplantation from animals, such as pigs, into humans (23). However, it took more than 20 years to discover that the expression of α-Gal raises another human threat.

Several observations, seemingly unrelated at first glance, needed to be threaded together to unravel the origin of an allergy to red meat, which was until then rarely diagnosed and not well understood. Since the 1990s, unpublished case reports described delayed allergic reactions several hours after eating red meat in the US, but no allergen could be identified (24). Several years later, in 2005, during a clinical trial for colon cancer patients, observations were made of acute and severe reactions upon the initial injection of cetuximab, a novel therapeutic monoclonal antibody against the epidermal growth factor receptor (EGFR) (7). Being on the market, cetuximab caused severe reactions in over 20% of patients treated for colon cancer or head and neck cancer in the southeast region of the US (25). In contrast, rates of hypersensitivity reactions were below 1% in the northeast region of the US.

In 2007, investigations by Chung et al. showed that cetuximab produced in Chinese Hamster Ovary (CHO) cells did not bind IgE in serum from anaphylactic cancer patients, while cetuximab produced in the Sp2/0 mouse cell line did, even though the amino acid sequence and binding affinity were the same. Hence, post-translational modifications might be the epitope for the pre-existing IgE, which often relate to glycosylation. Analysis of sera from patients reacting to cetuximab found pre-existing IgE specific for the oligosaccharide α-Gal. In addition, the prevalence of anti-α-Gal IgE antibodies was regionally distributed in a pattern consistent with the cases of cetuximab adverse effects (26). Indeed, a study showed that the variable portion of the heavy chain of the cetuximab antibody was consistently glycosylated with α-Gal (27).

Several patients sensitized to α-Gal and showing a hypersensitivity reaction towards cetuximab also reported adverse effects such as severe angioedema and anaphylaxis after eating pork. Commins et al. connected the dots of the first cases of delayed idiopathic reactions after red meat consumption with the reactions towards cetuximab, and described the first cases of delayed anaphylaxis or urticaria to red meat in patients with IgE to α-Gal (28), now known as AGS.

Already in 2002, it was reported that the majority of children tested in a rural Kenyan village showed IgE antibodies against cat allergens, without significant exposure to cats and without positive skin tests (29). Interestingly, in Sweden, patients with cat allergy had IgE antibodies against a carbohydrate epitope on cat IgA (30). Later, it was found that this oligosaccharide is indeed α-Gal (31). Equally, the unexplained predominance of IgE to cat allergens in the Kenyan village turned out to be almost entirely due to IgE to α-Gal (32).

In the following years, immunological identifications of relevant α-Gal-carrying allergens were carried out using AGS patient sera: bovine gelatin (33, 34), bovine laminin and collagen (35), porcine aminopeptidase N and angiotensin I converting enzyme (36), among others, were considered as possible triggers of allergic reactions to red meat in the context of AGS.

However, α-Gal is not only present on glycoproteins, but also on glycolipids, both of which are related to basophil activation in patients (37–39). While the clinical mystery about delayed allergic reactions to red meat has been largely attributed to a delayed intestinal absorption of α-Gal-carrying glycolipids and highly stable glycoproteins, and many investigators around the world started digging into different aspects of this quite intriguing type of allergy, an essential key point for understanding the etiology of AGS was to determine and investigate the source of the IgE sensitization to α-Gal.

## 3 The vector link

All humans naturally produce IgG, IgM and IgA antibodies reactive to the α-Gal oligosaccharide or α-Gal-like glycans. These polyclonal antibodies can have a broad specificity, depending on a person’s blood type (40). In fact, anti-α-Gal antibodies represent one of the most abundant antibody specificities in humans, accounting for roughly 1% of circulating immunoglobulins (19). Their continuous production is driven by exposure to carbohydrate antigens expressed by commensal gastrointestinal bacteria and dietary sources. The evolutionary loss of α-galactosyltransferase in humans, apes and Old World monkeys is thought to have conferred a selective advantage by enhancing immune recognition of pathogens that express α-Gal. Indeed, α-Gal or α-Gal-like glycans are present on several parasites and bacteria, including *Leishmania* spp. (9, 41), *Trypanosoma cruzi* (41), *Plasmodium* spp. (8), *Mycobacterium* spp. (42) and members of the Enterobacteriaceae (43). Correspondingly, α-Gal antibodies have been shown to reduce the burden of such pathogens in experimental models (8, 9, 44, 45). Conversely, the absence of α-Gal glycosylation on IgG molecules enhances antibody effector function and promotes protection against bacterial sepsis independently of α-Gal recognition (46). Nevertheless, the presence of the genuine α-Gal epitope, defined as Gal-α1,3-Gal, on parasites and bacteria remains debated, considering the use of tools with broader specificity, such as polyclonal, cross-reactive anti-α-Gal antibodies and lectins as demonstration for the α-Gal epitope expression (47). Despite the ubiquity and protective potential of α-Gal immunoglobulins, only a subset of individuals develops IgE antibodies directed against α-Gal. Yet, the underlying cause of this unusual sensitization pattern was initially not clear.

### 3.1 Ticks as a sensitization pathway

Scientists observed that hypersensitivity reactions to cetuximab occurred predominately at medical centers in the southeastern states, including North Carolina, Tennessee, Arkansas, Missouri and Virginia, whereas markedly fewer reactions were documented in northeastern cities such as New York, Boston or Chicago (25, 26). This striking regional pattern suggested that sensitization was linked to a geographically restricted environmental exposure. Indeed, the distribution of cetuximab reactions closely paralleled the incidence map of Rocky Mountain Spotted Fever (RMSF), a tick-borne infection caused by *Rickettsia amblyommatis* (7). Starting in 2007, van Nunen et al. mentioned for the first time clinical evidence for a connection between tick bites and delayed anaphylaxis to red meat: among 25 affected patients, 24 reported frequent tick bites and residence in a highly tick-endemic area near Sydney (48, 49). Shortly thereafter, Commins et al. demonstrated that tick bites can induce IgE antibodies to α-Gal (50). Subsequent case reports from Europe, Asia, and Central America further substantiated this link, showing that red meat allergy and α-Gal sensitization commonly follow, or are associated with, a history of tick bites (51–57).

As evidence accumulated across continents, it became clear that the ability to induce α-Gal-specific IgE is not restricted to a single tick species. Rather, across regions, a diverse set of ticks has been associated with α-Gal IgE formation or AGS. In the United States, *Amblyomma americanum* (*A. americanum*) has been identified as the primary sensitizing species, and more recently, bites from *Ixodes scapularis* (*I. scapularis)* have also been linked to α-Gal sensitization (50, 58). In Europe, *Ixodes ricinus* (*I. ricinus*) is the predominant species identified as vector (54). In Australia, both *Ixodes holocyclus* (*I. holocyclus*) and *Ixodes australensis* (*I. australensis*) have been implicated in red meat allergy and α-Gal-specific IgE formation (48, 59). In Japan, sensitization has been associated with bites from *Haemaphysalis longicornis* (*H. longicornis*) *and Amblyomma testudinarium* (*A. testudinarium*) (60, 61), while in South America, *Amblyomma sculptum* (*A. sculptum*) has been identified as a relevant vector (62). Several other tick species are suspected to be associated with AGS, yet robust mechanistic and epidemiological evidence remains limited (**Table 1**) (63).

**Table 1:** Overview of tick species evaluated for AGS association, α-Gal expression and experimental mouse sensitization.

Tick species	Region	AGS association	α-Gal detection	Tick stage*	Detection method	Used antibody	GT-KO mice sensitization	Exposure method	Key references
*A. americanum*	US	✔	✔	Adults	WB, IF, N-glycan profiling, IP-LC-MS/MS, 2DE/WB/LC-MS/MS	α-Gal mAb, Human sera	✔	TE (larvae), TSGE (adults), Tick bite (nymphs)	(50, 65, 66, 101, 102, 104)
*A. testudinarium*	Japan	✔	?		–	–	?	–	(61)
*A. sculptum*	South America	~	✔	Adults	ELISA	α-Gal pAb, MOA lectin, Mouse sera	✔	Tick bite, saliva (adults)	(62)
*A. maculatum*	US	?	✘	Adults	WB, IF, N-glycan profiling	α-Gal mAb	✘	Tick bite (nymphs)	(65, 104)
*I. scapularis*	US	✔	✔	Adults	WB, IF, N-glycan profiling, ELISA	α-Gal mAb	?	–	(58, 65, 66)
*I. ricinus*	Europe	✔	✔	Larvae, Nymphs, Adults	WB, ELISA, IHC, 2DE/WB/LC-MS/MS	α-Gal mAb, human sera, α-Gal pAb, MOA lectin	?	–	(54, 64, 66, 67, 70)
*I. holocyclus*	Australia	✔	✔	Adults	IP-LC-MS/MS	α-Gal mAb	?	–	(48, 66)
*I. australensis*	Australia	✔	?		–	–	?	–	(59)
*H. longicornis*	Japan	✔	✔	Adults	WB	α-Gal mAb	?	–	(60)
*H. marginatum*	Europe	?	✔	Adults	2DE/WB/LC-MS/MS	α-Gal mAb, human sera	?	–	(69)
*R. bursa*	Europe	~	✔	Adults	ELISA, 2DE/WB/LC-MS/MS**	α-Gal mAb, human sera	?	–	(66, 69)
*D. variabilis*	US	?	✘	Adults	WB	α-Gal mAb	?	–	(65)

*Tick life stage(s) analyzed for α-Gal detection.

**R. microplus BME/CTVM23 tick cells used.

✔, confirmed; ~, suggested; ✘, not detected; ?, unknown; WB, Western Blot; IF, Immunofluorescence; IHC, Immunohistochemistry; IP-LC-MS/MS, Immunoprecipitation followed by mass spectrometry; 2DE/WB/LC-MS/MS, Two-dimensional electrophoresis followed by immunoblotting and mass spectrometry; ELISA, Enzyme-linked immunosorbent assay; mAb, monoclonal antibody (clone M86); pAb, polyclonal Ab; MOA, *Marasmius oreades* agglutinin; TE, Tick extract; TSGE, Tick salivary gland extract.

### 3.2 α-Gal in ticks: detection and potential origins

The link between tick exposure, α-Gal sensitization, and the development of AGS was supported by the first identification of the α-Gal epitope in the gastrointestinal tract of *I. ricinus* by Hamsten et al. (64). Since then, several additional tick species implicated in α-Gal sensitization have been shown to harbor the carbohydrate epitope in different tissues and extracts. α-Gal was reported in *H. longicornis* salivary gland protein extract (60), and α-Gal-containing epitopes were later confirmed in the saliva of *A. sculptum* (62). Likewise, α-Gal was identified in the salivary glands and saliva of *A. americanum* and *I. scapularis*, but not in the midgut, with immunolocalization studies revealing α-Gal-bearing antigens adjacent to secretory vesicles, suggesting active secretion (65).

Proteomic analyses have shown that approximately 21-26% of salivary gland proteins and 15% of salivary proteins in ticks carry α-Gal modifications (66). Identified α-Gal-bearing proteins include vitellogenins, serpins, actin, α-2-macroglobulin, and members of the 14-3–3 chaperone family (66, 67). Apart from the identification of α-Gal-bearing proteins, a study pinpointed towards the presence of lipid-bound α-Gal in *A. americanum* (68). Interestingly, α-Gal-containing proteins were also found in tick species not yet clearly associated with AGS, such as *R. bursa* or *H. marginatum* (69), whereas other species, including *A. maculatum* and *D. variabilis*, lacked detectable α-Gal epitopes (65, 69). These observations indicate that the mere presence of α-Gal-modified glycoproteins in tick saliva is not sufficient to trigger sensitization, and that additional vector- or host-specific factors likely determine pathogenic potential.

Despite the consistent detection of α-Gal epitopes in tick tissues and secretions, the molecular origin remains unresolved. Three major hypotheses have been proposed: (i) acquisition from the host blood meal, (ii) endogenous synthesis by the tick, and (iii) contribution by the tick microbiome. Therefore, analyses of the spatial distribution of α-Gal in ticks and of various feeding stages were performed to further elucidate its origin. Spatial analyses revealed α-Gal epitopes within the midgut, hemolymph, and salivary glands of *I. ricinus*, colocalizing with clathrin-coated endosomes in the lumen and digestive gut cells. This finding suggests that α-Gal-bearing molecules from the host blood are internalized by endocytosis during hematophagy and subsequently processed in the midgut epithelium (70). Supporting this, α-Gal-containing molecules were upregulated after *A. americanum* feeding, whereas unfed ticks showed no detectable α-Gal in their salivary glands. Intriguingly, the expression appeared time-dependent across the feeding cycle, implying that α-Gal production or acquisition is feeding-driven (65, 71). Moreover, N-glycosylation structures increased over time after the onset of blood feeding in the salivary glands of *A. americanum* (72). Nevertheless, other tick species such as *I. scapularis* and *I. ricinus* presented α-Gal-containing antigens even in the unfed state (65, 70). Likewise, α-Gal epitopes have also been reported in the larval stage of *I. ricinus*, not yet having had a first blood meal (67). Similarly, ticks artificially fed on human blood, lacking α-Gal, still showed α-Gal-positive salivary glands (65). One study even reported higher α-Gal levels in salivary glands of ticks fed on human blood compared with other mammalian blood (73). While these findings argue against a simple passive acquisition from α-Gal-rich host blood, they have prompted alternative explanations. The presence of α-Gal in unfed ticks or those fed on α-Gal-free blood could reflect remnants from a previous life stage or *de novo* synthesis. One speculative hypothesis proposed that ticks might use a fucosidase to cleave fucose from human type B blood antigens, generating α-Gal antigens (65). However, this mechanism alone is unlikely to account for the high α-Gal levels observed in ticks fed on human blood. Collectively, these findings suggest that α-Gal expression in ticks is not solely dependent on the host blood source.

Alternatively, ticks may synthesize α-Gal through endogenous galactosyltransferases. Although the α-1,3-galactosyltransferase ortholog gene has not been identified in ticks, other galactosyltransferases, such as α-1,4- and β-1,4-galactosyltransferase, have been proposed to contribute to α-Gal synthesis (74, 75). However, silencing β-1,4-galactosyltransferase in *A. americanum* did not significantly reduce α-Gal levels in salivary glands, suggesting it is not a major enzyme in α-Gal biosynthesis. In contrast, silencing of α-D-galactosidase resulted in reduced α-Gal-containing molecules, indicating that this enzyme may release α-Gal from host-derived substrates. In fact, its differential expression across the feeding stages supports a role in remodeling host glycoconjugates (75).

A third possible source is the tick microbiota. Tick-borne pathogens such as *Anaplasma phagocytophilum* and *Borrelia burgdorferi* sensu lato have been shown to increase tick α-Gal levels or to express α-Gal themselves (74, 76). Another interesting aspect is the possible involvement of the α-1,3-galactosyltransferase from the tick microbiome. Indeed, α-1,3-galactosyltransferase activity and the formation of α-Gal structures on proteins have been documented in tick-associated bacteria, supporting a potential microbial contribution (43, 77, 78). However, to date, no established association between α-Gal sensitization and any known tick-borne disease has been demonstrated.

Regardless of its precise origin, α-Gal is consistently found in the intestinal tract and saliva of several tick species. This provides a plausible molecular link between tick bites and the cutaneous sensitization to α-Gal-containing antigens, ultimately leading to IgE-mediated allergic responses upon ingestion of mammalian meat. However, the mechanisms by which skin exposure to tick-derived α-Gal induces and maintains IgE production in humans remain largely unexplored.

## 4 Sensitization: how does the immune system respond?

**Figure 1:**
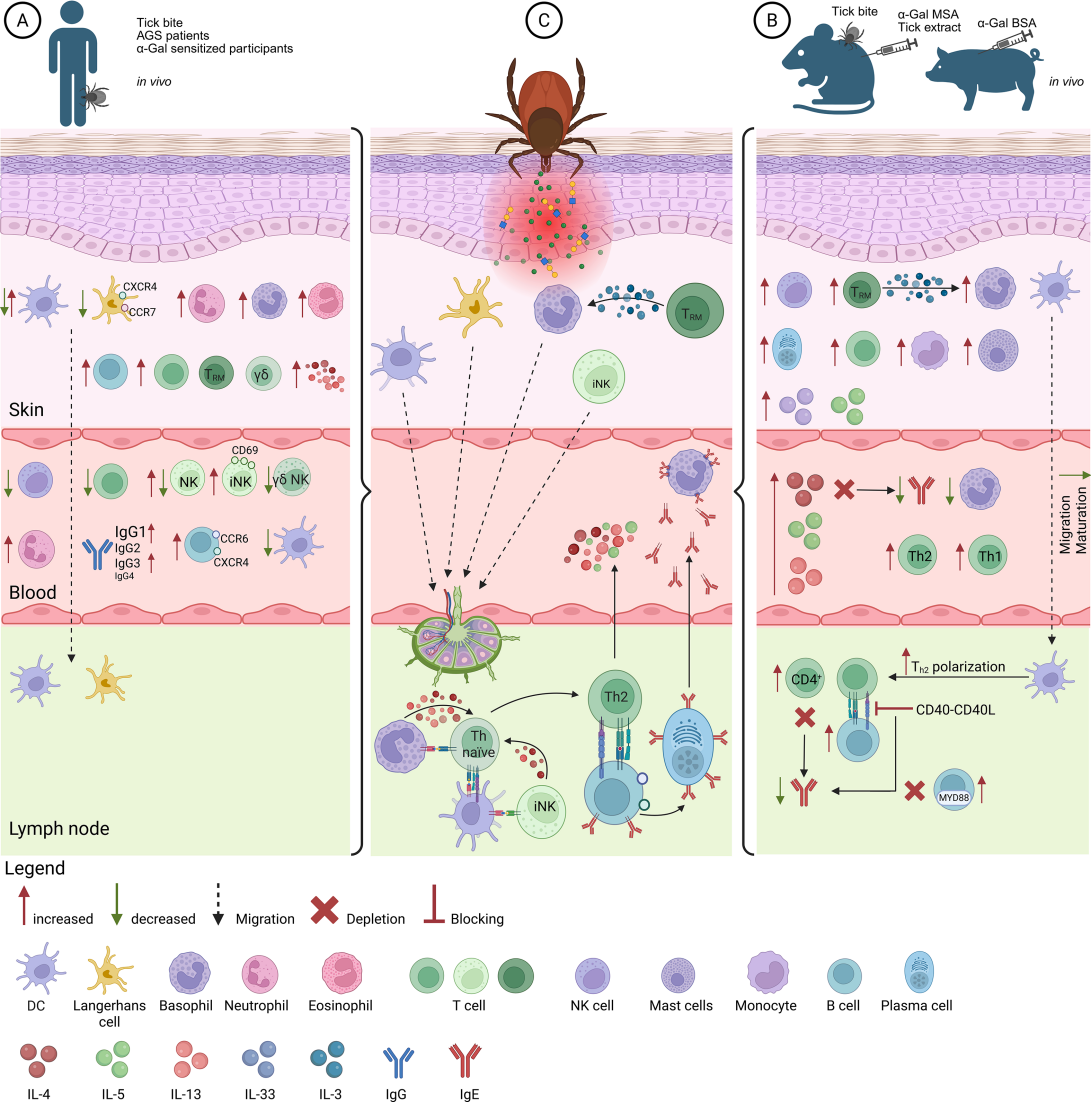
Integrated overview of human and animal immune phenotping associated with α-Gal sensitization, along with a proposed mechanism underlying the sensitization process. **(A)** Descriptive immune phenotypes observed in tick-exposed, α-Gal-sensitized, or AGS individuals, summarizing changes reported in skin, blood, and lymphoid tissues across observational studies. **(B)** Corresponding immune phenotypes in rodent and pig models that were experimentally sensitized via tick bites or by inoculation with α-Gal carriers (α-Gal-MSA, α-Gal-BSA), integrating descriptive observations with mechanistic insights obtained through targeted interventions. **(C)** Conceptual model of early sensitization events at the tick bite site, integrating convergent observations from human and animal studies. The panel depicts a plausible sequence of immune interactions leading to α-Gal-specific IgE induction. Created in BioRender. Petry, J. (2026) https://BioRender.com/s8n92ul.

Therapeutic options for AGS remain limited to strict avoidance of mammalian-derived products, prevention of further tick bites, and symptomatic management. To develop targeted interventions, deeper insight into the mechanisms driving α-Gal sensitization is urgently needed. Despite increasing awareness of AGS, our understanding of the immune responses to tick bites and the subsequent sensitization process remains incomplete. A central question is why tick exposure so effectively induces IgE responses to α-Gal, whereas continuous oral or microbial exposure to the same carbohydrate epitope typically promotes tolerance. This suggests that the cutaneous route of exposure, together with immunomodulatory factors in tick saliva, as for instance PGE_2_, provides a unique context favoring Th2 polarization and IgE class switching. Likewise, not all individuals exposed to ticks develop α-Gal sensitization or AGS, indicating that host-specific susceptibility factors or tick-specific conditions may critically influence the outcome. Interestingly, other hematophagous arthropods such as mosquitoes or bedbugs do not appear to elicit α-Gal-specific IgE, whereas chiggers and cat fleas might be implicated, further underscoring the unique immunological imprint of tick bites (79, 80). Immunological studies addressing these processes have relied on diverse experimental systems, ranging from human cohorts and *ex vivo* samples to animal models of sensitization, and have examined both local and systemic immune alterations (**Figure 1**). In the following section, we integrate these findings across models to delineate the cellular and molecular events that connect tick feeding to α-Gal-specific IgE induction and allergic sensitization.

### 4.1 Human studies

To better understand the sensitization process, research has examined skin biopsies and peripheral blood samples, typically from individuals with a history of tick exposure, regardless of the α-Gal sensitization or AGS status of the individuals (**Figure 1A**).

#### 4.1.1 Immune response to tick bites

The immunological consequences of tick feeding and the accompanying barrier disruption for local and systemic immunity remain incompletely defined, mainly due to limited studies in human skin. Nevertheless, several groups have analyzed immune cell populations in the skin and blood of tick-bitten individuals.

Following tick attachment, skin biopsies revealed pronounced changes in local immune composition. Notably, tick bite lesions initiate early innate immune activation, reflected by elevated mRNA expression of markers and chemoattractants for macrophages, neutrophils, and dendritic cells (DCs), particularly within the first 24 hours. A subsequent, though less pronounced, adaptive response followed, characterized by increased mRNA levels for CD4^+^ and CD8^+^ T cells and lymphocyte chemoattractants (81). Correspondingly, bulk transcriptomic analysis of skin biopsies revealed enrichment of IL-17, peroxisome proliferator-activated receptor (PPAR), and AMP-activated protein kinase (AMPK) signaling pathways, suggesting coordinated activation of inflammatory and metabolic programs that may reflect both immune defense and tissue repair (82). Isolation of leukocytes from tick-bitten skin revealed increased numbers of neutrophils, B cells and T cells, including tissue-resident memory T cells (T_RM_) and γδT cells, while decreased counts of Langerhans cells and dermal DCs compared to those isolated from non-affected healthy skin (83). In contrast, artificial skin punctures showed no such effects, suggesting that the immunomodulatory patterns are induced by tick feeding and not by barrier disruption (83). Correspondingly, tick bites reprogram Langerhans cells to increase migration into lymphatic tissues by upregulating CXCR4 and CCR7 (84). Moreover, significantly higher basophil counts were found at the site of tick bites compared to adjacent skin areas, but only after 12 hours of attachment (85).

Taking into account the α-Gal sensitization after tick bites, studies revealed an increasing infiltration of basophil and eosinophil numbers in skin lesions from individuals being sensitized against α-Gal and reporting a history of multiple tick bites (61, 86). Concomitant with other publications on tick bites only, CD4^+^ T cell infiltration was significantly greater in the skin lesions of α-Gal-sensitized individuals with a history of multiple tick bites (61). Notably, basophils can drive Th2 polarization by secreting IL-4 and IL-13 and functioning as antigen-presenting cells (87, 88). In line, differentially expressed gene analysis from tick-bitten skin and healthy skin biopsies revealed an enriched IL4- and IL-13 signaling pathway in patients bitten by a tick (89). These findings raise the possibility that repeated tick exposure triggers basophil-driven Th2 skewing, potentially leading to B cell class switching and the production of anti-α-Gal IgE antibodies. Interestingly, even though elevated numbers of T cell subsets were found in the skin after a tick bite (61, 83), impaired T cell responses were measured *ex vivo* upon stimulation, resulting in a decreased ratio between Th1/Th2 skin-infiltrating T cells (61, 83, 86).

Bulk sequencing of peripheral blood from tick-bitten individuals revealed differential expression of genes related to platelet aggregation, extracellular exosomes, and immunoglobulin receptor binding, alongside upregulation of immune activation genes in T cells, macrophages, and neutrophils (82). Phenotyping of isolated leukocytes in peripheral blood upon a tick bite showed a reduction in natural killer (NK) cells, T cells, NK T cells and type 3 innate lymphoid cells were observed, while neutrophil counts were increased (83).

Together, these studies provide a comprehensive first glimpse into how tick bites reshape the immunological landscape of both skin and circulation. However, most investigations did not assess α-Gal sensitization status, making it difficult to establish direct correlations between immune cell alterations and IgE development.

#### 4.1.2 Immune response in α-Gal sensitization and AGS

Following the general immune perturbations caused by tick bites, studies on AGS patients have provided insights into systemic immune regulation associated with α-Gal sensitization. Most human data derive from peripheral blood analyses, as skin-based studies in AGS patients are limited.

Serological studies have shown that the IgG subclass distribution against α-Gal markedly differs from that observed in typical protein-based allergens or in non-allergic individuals. In AGS patients, IgG1 predominates, followed by IgG2 and IgG3, whereas IgG4 levels remain low (90–92). A similar IgG subclass pattern was found in a high-risk cohort of α-Gal-sensitized and non-sensitized forestry workers with recurrent tick exposure (93). Several studies showed that both AGS patients and sensitized individuals display higher IgG1 and IgG3 levels than non-sensitized controls (91, 93, 94). Nevertheless, the predominance of IgG1 is not unique to α-Gal but reflects a typical feature of anti-carbohydrate allergic responses. Individuals reacting to cross-reactive carbohydrate determinants exhibit a comparable IgG subclass profile (90). Notably, non-sensitized but tick-exposed forest employees showed elevated α-Gal-specific IgG1, suggesting that recurrent tick bites can stimulate an IgG1-dominant response independent of the sensitization status (93). By contrast, fish- or birch pollen-allergic individuals typically produced IgG4-dominant responses, with lower IgG1 and IgG2 levels against the major salmon, cod, or apple allergen (90, 91, 93). Furthermore, in α-Gal-non-sensitized individuals, IgG2 predominates (92–95), possibly reflecting immune responses to gut microbiota expressing α-Gal (22, 92, 93), although the presence of the genuine α-Gal epitope, defined as Gal-α1,3-Gal, on commensal gut bacteria remains debated (47). Overall, this atypical subclass distribution distinguishes AGS and α-Gal-sensitized individuals from classical food allergies and from natural α-Gal immunity in non-allergic subjects, highlighting that different immune mechanisms might be involved in predisposed individuals.

Given their role as the source of antibody production, B cells represent a critical component of α-Gal sensitization. However, the specific B cell subsets responsible for generating α-Gal-specific IgE remain incompletely defined. While AGS patients and healthy controls generally exhibit a similar distribution of major circulating B cell populations, including naïve, unswitched memory, switched memory, and double-negative B cells (96–98), B cell populations distinct from the classical isotype-switched memory B cells were reported. In red meat-allergic subjects, these B cell clusters displayed higher IgD and lower IgM expressions, along with increased chemokine receptor CXCR4 and CCR6 levels (96). A subsequent study confirmed that CCR6^+^ class-switched memory B cells with high CXCR4 expression were significantly enriched in sensitized individuals in contrast to non-sensitized individuals (97). Moreover, these B cell subsets were indeed capable upon *in vitro* stimulation to produce anti-α-Gal IgE (96). A very recent study, analyzing B cells from AGS patients via scRNA sequencing, confirmed a cluster of α-Gal-specific IgE-secreting B cells, including CCR6-proficient B cells, however, CCR6-deficient plasmablast/plasma cells (98). Activation of the CCL20/CCR6 and CXCL12/CXCR4 pathways further enhanced IgE isotype switching and proliferation (97). In contrast, after a tick bite, AGS subjects exhibited increased frequencies of major B cell parent and subpopulations, including CD27^-^IgD^-^ double-negative cells and CD19^+^CD20^-^HLA-DR^+^ plasma cells, compared to healthy donors (99). Moreover, the transcriptional profiling of B cells in PBMCs revealed distinct gene expression patterns separating α-Gal-sensitized from non-sensitized individuals (100). These findings point to novel B cell signatures associated with α-Gal-specific IgE production and might play a role in the pathogenesis of this food allergy.

Comparable to B cells, transcriptional immune profiling of peripheral T cells has distinguished sensitized from non-sensitized individuals, revealing clusters of differentially expressed genes associated with antigen processing and presentation, such as *HLA-DRB3*, *HLA-DMA*, and *CD83*, as well as type 2 immune responses, like *IL13A*, *VEGFA*, *ICAM1*, or *CD74* (100). Given the fact that the α-Gal epitope can also be expressed on glycolipids, it is plausible that lipid antigens within tick saliva might engage invariant NK T (iNKT) cells, which recognize glycolipid antigens presented by CD1d. In support of this, increased frequencies of circulating activated CD69^+^ iNKT cells were detected in sensitized individuals compared to controls, although this difference did not reach statistical significance. Interestingly, iNKT cell numbers positively correlated with α-Gal-specific IgE levels (100). Similarly, enrichment of NKT cell clusters was reported in AGS subjects, though the iNKT subset was not specifically determined (98). Conversely, a significant decrease in NKT cells, particularly γδ NKT cells, was observed in AGS patients shortly after a tick bite compared to healthy donors (99). However, this analysis lacked phenotypic resolution for activation markers and invariant subsets. Finally, scRNA analyses identified a rare NKB-like cell population in AGS patients expressing CD1A and CD1C transcripts, suggesting potential capacity to recognize α-Gal-bearing glycolipid antigens (98). Activation and polarization of naïve CD4^+^ T cells are dependent on the antigen presentation of DCs. Notably, in AGS patients, reduced frequencies of circulating DCs and plasmacytoid DCs (pDCs) have been observed following tick bites compared with controls (99). Unfortunately, detailed phenotypic and functional analyses of human DC subsets in AGS are still lacking.

Investigating the sensitization mechanism to α-Gal directly in AGS patients remains challenging, as these individuals are already sensitized, and longitudinal studies on tick bites preceding sensitization are missing. Consequently, experimental animal models are essential to dissect the early immunological events leading to α-Gal-specific IgE production.

### 4.2 Rodent models

Given the ethical limitations of exposing humans to tick bites or collecting skin biopsies, animal models have become indispensable for investigating the immunological mechanisms underlying sensitization to α-Gal. Human studies on AGS patients or tick-bitten individuals are limited, as sensitization has already occurred and baseline immune states prior to exposure are unknown. To overcome this, a murine model deficient in the α1,3-galactosyltransferase gene (GT-KO) was established, resulting in the inability to synthesize α-Gal epitopes. Upon exposure to tick bites, tick-derived material or α-Gal-containing proteins, these mice generated indeed α-Gal-specific IgE and showed subsequent reactions towards α-Gal challenges (62, 101–103) (**Figure 1B**, **Table 1**).

#### 4.2.1 Immune response to tick bites

Biopsies from tick bite sites in sensitized and non-sensitized α-Gal-deficient mice analyzed by mRNA gene expression profiling revealed a predominance of macrophages and NK cells during initial infestation. Upon secondary tick exposure in α-Gal-deficient mice, transcriptional signatures indicated a shift from innate to adaptive immune responses (104). Although early infestations in wild-type mice expressing α-Gal did not induce detectable basophil infiltration (105, 106), CD4^+^ T_RM_ cells were identified in distant, non-infected skin areas (106). However, following a second tick bite in wild-type mice, a pronounced basophil accumulation appeared at the tick feeding site (105, 106), which was highly dependent on IL-3-secreting CD4^+^ T cells exhibiting resident and memory phenotypes, found earlier in the non-infested skin (106). This basophil recruitment is a hallmark of acquired tick resistance (ATR), a phenomenon in several mammalian species, such as mice and guinea pigs characterized by reduced tick engorgement, premature detachment and sometimes death of the parasite (107). Diphtheria toxin-mediated basophil depletion in *Mcpt8^DTR^* mice abolished ATR, underscoring the central role of basophil infiltration after second tick infestation (105, 107). Thus, basophil infiltration is clearly critical for ATR and its role might also be relevant in the context of α-Gal sensitization, although a direct role has not yet been demonstrated.

#### 4.2.2 Immune response to tick extract, tick saliva and α-Gal-carrying proteins

##### 4.2.2.1 Tick extract

Inoculation of the whole tick extract (TE) demonstrated the route-dependent nature of sensitization. Mice sensitized intraperitoneally failed to develop tick-specific IgE, whereas subcutaneous exposure induced α-Gal-specific IgE (101). Moreover, examination of the skin after TE exposure showed a mixed inflammatory infiltrate, including granulocytes, lymphocytes and plasma cells. Within draining lymph nodes, germinal center B cells and T follicular helper (Tfh) cells increased following cutaneous exposure (101). Depletion of CD4^+^ T cells abolished IgE but not IgG responses, underscoring the requirement for T helper cells and Th2 cytokines for class switching. Blocking CD40-CD40L interactions during sensitization similarly prevented IgE induction, confirming the necessity of T cell help. Mechanistically, whole TE, without any use of additional adjuvants, activates Toll-like receptor (TLR) 2, TLR4, TLR5 and TLR9, all signal via myeloid differentiation primary response 88 (MyD88). Subsequently, mice lacking MyD88 specifically in B cells failed to induce IgE responses, demonstrating that MyD88-dependent signaling in a B cell intrinsic manner is essential for class switch recombination to IgE (101).

##### 4.2.2.2 Tick saliva

*In vivo* exposure to tick saliva was found to impair the maturation of DCs and limit their early migration from inflamed skin to draining lymph nodes. Moreover, DCs showed a reduced capacity to present soluble antigen to T cells (108).

##### 4.2.2.3 α-Gal-carrying proteins

Given that tick extracts contain numerous non-specific proteins and lipids, Hils et al. used defined α-Gal-carrying conjugates to dissect the immune response more precisely. Subcutaneous sensitization of GT-KO mice with α-Gal bound to mouse serum albumin (MSA) or human serum albumin (HSA), in combination with aluminum hydroxide, induced local infiltration of basophils, eosinophils, and mast cells at the sensitization site. In skin-draining lymph nodes, α-Gal-specific B cells, germinal center B cells and IgE^+^/IgG1^+^ B cells were detected (103).

Serologically, sensitized mice exhibited elevated IL-4, IL-5 and IL-13 levels, confirming a type 2 cytokine milieu. Notably, IL-4 depletion reduced basophil recruitment, prevented IgE production, and protected mice from anaphylaxis upon α-Gal challenge (103). However, basophil depletion alone did not rescue mice from anaphylaxis (103).

### 4.3 Non-rodent models

#### 4.3.1 Pig model

To complement rodent studies, α1,3-galactosyltransferase knockout pigs have been generated. These animals, originally developed to study α-Gal-mediated hyperacute rejection in xenotransplantation (109, 110), provide a physiologically relevant model for investigating α-Gal sensitization. Compared with rodents, porcine skin more closely resembles human skin in its structure, making it particularly suitable for studying cutaneous sensitization mechanisms. Subcutaneous injections of α-Gal-bovine serum albumin (BSA) induced elevated levels of α-Gal-specific IgE. Analysis of sensitized skin revealed an increased presence of T cells, particularly Th1, Th2 and regulatory T (Treg) cells, as well as monocytes and mast cells. In parallel, mRNA levels of type 2 immune mediators IL-33, IL-5 and thymic stromal lymphopoietin (TSLP) were significantly upregulated in sensitized skin. At the systemic level, sensitized pigs showed higher frequencies of circulating Th1 and Th2 cells compared with non-sensitized controls (111) (**Figure 1B**).

#### 4.3.2 Zebrafish model

Zebrafish are widely used in toxicology, inflammation, and oncology research, but only recently explored as a model for allergic diseases, including AGS. Unlike rodents or pigs, zebrafish naturally lack endogenous α-Gal expression, eliminating the need for α1,3-galactosyltransferase knockout lines. Nevertheless, zebrafish produce anti-α-Gal IgM antibodies likely due to commensal gut microbiota (112). This feature has prompted their use to investigate immune mechanisms triggered by tick salivary components and mammalian meat consumption in the context of AGS.

Following intramuscular or subcutaneous sensitization with tick saliva or with α-Gal-BSA, zebrafish displayed elevated anti-α-Gal IgM levels (112–114). However, deglycosylated tick saliva also induced anti-tick IgM (115), suggesting that antibody responses may target salivary proteins independently of α-Gal. Upon oral challenge with mammalian meat (dog food), sensitized zebrafish developed hemorrhagic anaphylactic-type reactions and behavioral changes such as reduced mobility and erratic swimming (112–114). These reactions were associated with tissue-specific TLR-dependent Th1 responses in the intestine and Th2 responses in the kidney as measured by mRNA levels (112, 116). Multi-omics profiling of the gut revealed downregulation of pathways related to blood circulation, smooth muscle contraction and immune signaling (113, 117). In parallel, tick saliva sensitization and mammalian food exposure altered gut microbial composition, potentially through antibody-mediated opsonization of α-Gal-expressing bacteria (118). Notably, severe manifestations occurred in some zebrafish upon injections with tick saliva and even deglycosylated saliva alone, but not with α-Gal-BSA, without the need for mammalian meat challenge. This indicates that tick salivary proteins can elicit strong α-Gal-independent reactions (112, 115). Proteomic analyses identified key allergenic components in tick saliva, such as antigen p23 and metalloproteases lacking α-Gal modifications (115). Subsequent injections of these recombinant molecules together with salivary non-protein fractions reproduced similar responses (116). Lipidomic profiling of tick saliva revealed high levels of phosphatidylcholine and diacylglycerol. Importantly, measuring anti-phosphatidylcholine in the serum of AGS patients indeed revealed a significant association with tick bites, supporting a possible role for tick salivary lipids without α-Gal modifications in AGS (117).

Despite these insights, the zebrafish model has significant limitations. Only three immunoglobulin isotypes (IgM, IgD, IgT/Z) are described in these fish, and no IgE analogue exists (119). Moreover, due to their less complex adaptive immune system, lack of FcϵRI receptor and poorly characterized eosinophils and basophils (120), this model has limited capacity to study the mechanism of skin-driven α-Gal IgE sensitization.

### 4.4 *In vitro* models

**Figure 2:**
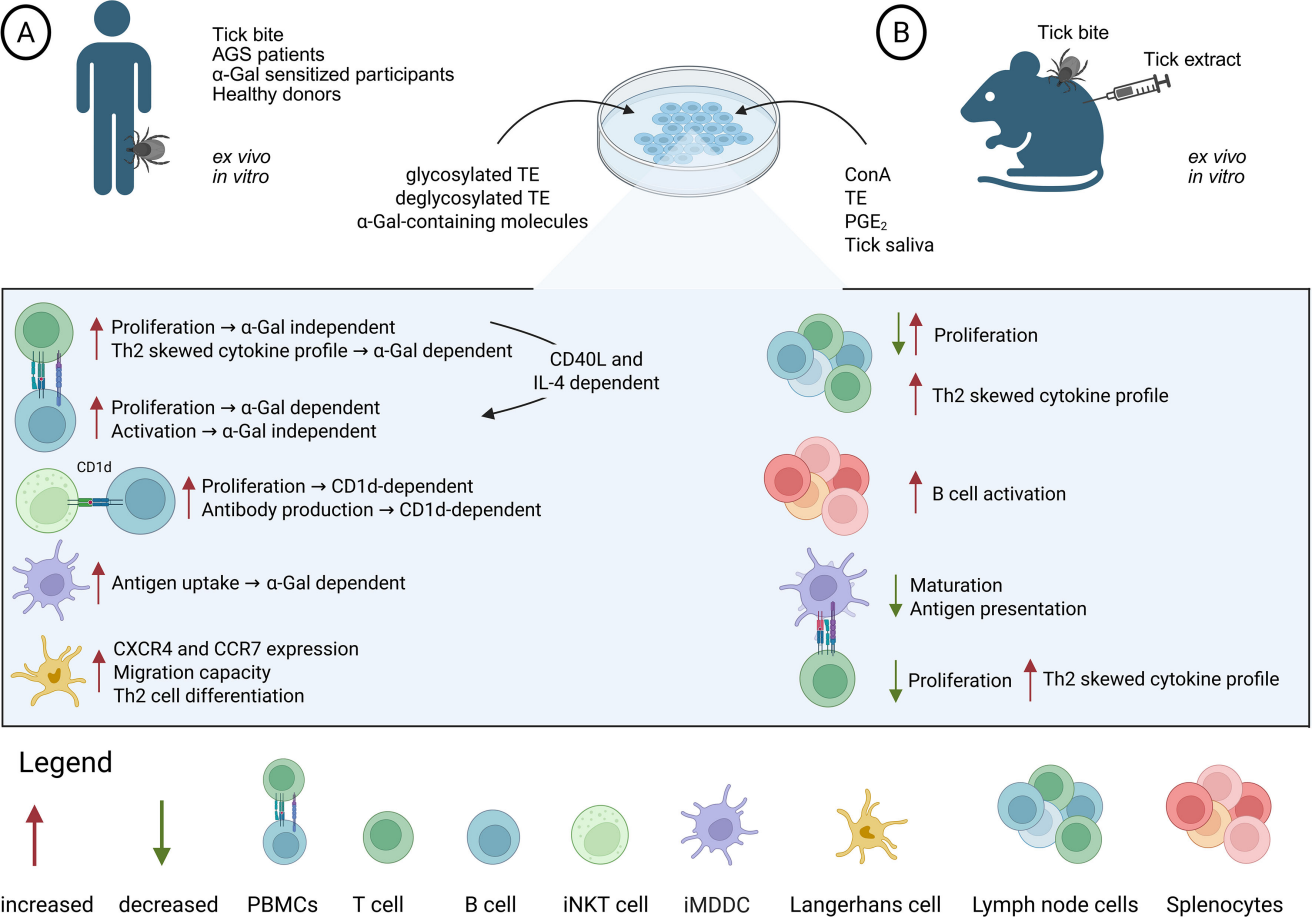
*In vitro* and *ex vivo* immune responses of human and murine cells to stimuli. **(A)** Analyses of human immune cells from tick-exposed or α-Gal-sensitized subjects, illustrating proliferative responses, cytokine profiles, antigen uptake, and activation patterns elicited by different stimuli. **(B)** Analyses of murine immune cells collected from tick-infested or α-Gal-sensitized animals, highlighting alterations in proliferation, activation, antigen-presenting cell function, and Th2-associated cytokine profiles caused by different stimuli. Created in BioRender. Petry, J. (2026) https://BioRender.com/iddqd87.

Further mechanistic insights into α-Gal sensitization have been obtained through *in vitro* and *ex vivo* experiments, particularly using PBMCs from AGS patients or lymph node cells as well as splenocytes from sensitized mice. TE, tick saliva and salivary gland extracts have been shown to modulate multiple immune functions at different levels (**Figure 2**).

Although lymph nodes from tick-infested mice were three times larger than those from controls (121), *ex vivo* stimulation of these lymph node cells revealed a decreased proliferative response to concanavalin A (ConA) (121), whereas specific stimulation with tick salivary gland extract induced proliferation (122). Similarly, *ex vivo* stimulation of B and T cells from AGS patients with TE resulted in stronger proliferation compared to healthy donors, although only B cell proliferation was dependent on α-Gal glycosylation (123). Moreover, *ex vivo* stimulation of lymph node cells from tick-infested mice with ConA enhanced IL-4, IL-10 and TGF-β expression, while reducing IL-2, IFN-γ and IL-12 secretion, indicating a Th2-skewed cytokine profile (121, 122). Correspondingly, *ex vivo* stimulation of T cells from AGS patients with TE induced a pronounced Th2 cytokine pattern, dependent on the glycosylation state of the extract, whereas B cells upregulated the activation marker CD23 in an α-Gal-independent manner (123). Likewise, *ex vivo* stimulation of splenocytes with tick salivary gland extract increased the early activation marker CD69 on B cells (102). Notably, B cell proliferation and early activation were partly dependent on CD40L and IL-4, suggesting T cell-derived Th2-type help (123). The CD1d molecule, capable of displaying glycolipid antigens, is not only expressed on DCs, but also on B cells. Thus, iNKT cells could also provide direct help for B cell proliferation and anti-α-Gal IgE production. Indeed, in a T and B cell co-culture, B cell proliferation and α-Gal glycolipid-induced antibody production were inhibited by anti-CD1d, demonstrating that these responses depended on CD1d-restricted B cell-iNKT cell interactions (124).

To further dissect the mechanisms underlying Th2 polarization, DCs have been used as a key model to study the impact of tick saliva and α-Gal-carrying molecules on antigen presentation. In immature monocyte-derived DCs (iMDDCs), uptake of α-Gal-BSA occurred more efficiently than that of non-α-Gal-carrying BSA, via micropinocytosis or receptor-mediated endocytosis, indicating that α-Gal residues enhance antigen uptake and may affect subsequent antigen processing and presentation (125). In an artificial human skin model, it was shown that epidermal Langerhans cells decrease after injection of salivary gland extracts into human skin explants and upregulate CXCR4 and CCR7 expression, indicating cell migration. Indeed, stimulation of monocyte-derived LCs with salivary gland extracts showed higher CXCR4 and CCR7 expression and increased migration capacity toward collagen gel (84). Moreover, it has been shown that PGE_2_ present in tick saliva reduced the DC capacity to stimulate T cell proliferation and IL-2 production (126). In contrast, DCs pulsed *in vitro* with tick saliva promoted a Th2 differentiation of naïve CD4^+^ T cells in an IL-4-dependent manner (108, 127). Likewise, tick salivary gland extract-stimulated Langerhans cells induced a Th2 cell differentiation in a human splenic tissue spheroid culture model (84), showing the capacity of tick extracts to alter naïve T cells toward a Th2 polarization.

### 4.5 Summary

Integrating findings from human, murine, and other animal models reveals converging immunological mechanisms underlying tick bites and α-Gal sensitization, despite considerable variability. Differences likely arise from biological heterogeneity among individuals, such as sensitization levels, and among ticks, like species, developmental stage, bite duration, as well as methodological diversity in experimental models, sensitization routes, and α-Gal-containing molecules. Consequently, data across different models remains difficult to compare, given inconsistent exposure histories and sampling time points (**Figure 1C**).

In murine models, intradermal sensitization with tick saliva, TE or salivary gland extract consistently induced α-Gal-specific IgE production (62, 101–103). In contrast, virus-like particles carrying α-Gal or uncoupled α-Gal failed to elicit α-Gal-specific IgE, highlighting that α-Gal alone is insufficient and that a carrier protein is required, yet not every carrier works, implying that the nature of the carrier protein and/or adjuvant factors may be decisive for sensitization (62, 103). Moreover, α-Gal-bearing proteins required alum to induce antibody responses, whereas tick-derived preparations did not, indicating that tick saliva possesses intrinsic adjuvant activity. Indeed, TE activated multiple TLRs and exerted a potent adjuvant effect, as co-sensitization with unrelated antigens induced IgE responses comparable to those elicited with alum (101). The route of exposure proved equally important: subcutaneous, but not intraperitoneal, sensitization generated α-Gal-specific IgE (101).

At the APC level, findings are partly contradictory. Tick bites were reported to reduce dermal DC and Langerhans cell numbers (83, 84), yet others observed DC gene enrichment and increased DC presence in skin lesions (81). These discrepancies likely reflect temporal differences in sampling after tick detachment. Circulating DCs, particularly pDCs, were reduced in AGS patients (99). Given that pDC depletion promotes allergic airway sensitization (128), and that low pDC counts are associated with asthma development in children (129), a low pDC frequency could predispose to Th2 polarization. Alternatively, reduced peripheral and dermal DCs could indicate migration into lymphoid tissues. Indeed, tick bites and tick salivary gland extracts could reprogram Langerhans cells to enhance migration into lymphatic tissues (84), while tick saliva impaired skin DC migration to draining lymph nodes and antigen presentation (108, 126). Nevertheless, Th2 differentiation is fostered by tick bites and tick saliva *in vitro* as well as *in vivo* (84, 108, 127). Thus, DCs may act as both attenuated antigen presenters and active promoters of Th2 skewing in α-Gal sensitization. However, the mechanisms underlying these quantitative DC changes remain to be clarified.

Skin immune infiltrates in sensitized animal models and sensitized or tick-bitten humans are enriched in basophils, eosinophils and mast cells (61, 85, 86, 103, 105, 106). Basophils, in particular, accumulate at tick re-infestation or sensitization sites and are crucial for ATR (103, 105, 107). Notably, single-cell transcriptomic profiles of human tick-bite lesions closely resembled those of ATR skin guinea pigs (82, 130), suggesting that local erythema and pruritus in humans may represent manifestations of ATR (82, 130). Hence, it was suggested that the human α-Gal-specific IgE response may therefore reflect an evolutionary synonym of ATR in humans. Nevertheless, research investigating human ATR is still in the beginning, but merits higher attention, in order to better understand the tick-induced α-Gal sensitization. Notably, basophils may also contribute to Th2 skewing and B cell activation, as they can infiltrate lymph nodes and provide IL-4 (87, 88). Accordingly, basophil infiltration and basophil-T cell crosstalk following tick bites have been proposed to play a critical role in anti-tick immunity and to be a possible mechanism to skew a Th2 cell response and IgE production (131). Yet, basophil depletion did not abrogate α-Gal-specific IgE formation (103), suggesting a modulatory rather than indispensable role for basophils as an initial source of IL-4 in the early sensitization phase. However, these findings were obtained using a subcutaneous α-Gal-MSA and alum-based sensitization model, rather than a tick bite-induced AGS model, and therefore may not fully capture the role of basophils during natural cutaneous sensitization.

T cells are consistently expanded in skin and lymphoid tissues following tick exposure (61, 81, 83, 106, 111). Particular CD4^+^ T_RM_ cells are of interest, since they were essential for IL-3-mediated basophil recruitment and anti-tick immunity (83, 106). Interestingly, GATA-3, a hallmark of type 2 immunity, was shown to be abundant in effector and regulatory T cells of the skin (132). Notably, CD4^+^ T cells and IL-4 are indispensable for α-Gal-specific IgE production in GT-KO mouse models, as their depletion abrogated antibody generation and basophil accumulation (101, 103), indicating their critical role as a central driver of the Th2-biased milieu necessary for sensitization. Indeed, Th2 cytokine polarization after tick exposure or sensitization was consistently observed (100, 103, 111, 121–123). While promoting a Th2 cytokine profile was partly dependent on α-Gal, increased proliferation of T cells seemed to be independent of α-Gal stimulation (123), suggesting that other salivary proteins provide the proliferative signals. Apart from αβ T cells, unconventional T cell subsets may also shape sensitization. Sensitization of TCRβ-deficient mice with α-Gal-bearing pig cells revealed a dependence on αβ T cells and CD154 for IgM responses, while residual antibody production in TCRβ-deficient mice suggested compensatory roles for γδ T cells (133). Indeed, γδ T cells were increased in tick-bite lesions (83), but decreased in circulation immediately post-tick bite in AGS patients (99). Strikingly, γδ T cells have been documented to play a key role in immune surveillance of barrier tissue (134), which might indicate that particular γδ T cells infiltrate from blood to skin and might be implicated in the sensitization to α-Gal. Similarly, iNKT cells were slightly elevated and correlated with α-Gal-specific IgE levels (98, 100). Experimental blockade of CD1d impaired anti-α-Gal antibody production, supporting their involvement (124). As iNKT cells secrete IL-4, they may reinforce B cell class switch towards IgE (135). Nevertheless, the contribution of unconventional T cells, like γδ T cells and iNKT to AGS remains insufficiently characterized and warrants deeper investigation.

The molecular pathway guiding B cells from a naïve to an IgE-producing B cell after tick exposure remains incompletely understood. Two germinal center (GC)-dependent pathways have been proposed: (1) direct differentiation of GC-derived IgE B cells that originate from naïve IgM B cells into IgE-secreting plasma cells, or (2) sequential class switching from IgG1^+^ intermediates upon antigen restimulation (136). In addition, GC-independent IgE^+^ B cell responses have been described, although the mechanisms remain poorly understood (137). Given that α-Gal is a carbohydrate antigen, GC-independent IgE responses may be conceptually relevant, as carbohydrate antigens are known to engage T cell–independent B cell activation pathways (138). Following tick bites in humans and mice, B cell expansion occurred in skin, blood and lymph nodes (83, 99, 101, 103). TE stimulation enhanced B cell proliferation and activation through mechanisms partially dependent on α-Gal, CD154 and IL-4 (102, 122, 123). Consistently, α-Gal-specific IgE formation required CD4^+^ T cell help and IL-4 signaling, as both depletion of CD4+ T cells and blockade of CD154 impaired antibody production (101, 103). Tick proteins may also deliver intrinsic adjuvant signals via TLR-MyD88 pathways, as MyD88 deletion in B cells reduced α-Gal-specific IgE (101). Finally, distinct B cell clusters expressing CCR6 and CXCR4 were identified in AGS patients, capable of IgE class switching and α-Gal-specific secretion upon CCR6/CXCL4 axis activation (96–98), suggesting that specialized B cell programs may underlie this unique carbohydrate-specific allergy (**Figure 1C**).

## 5 Epidemiology, environmental exposure, and host susceptibility

Although AGS has been reported worldwide, estimating its true prevalence remains challenging due to under-diagnosis. Nevertheless, increasing numbers of population-based serologic screenings are revealing the prevalence of α-Gal-specific IgE and helping to clarify regional variations, risk factors, tick vectors and potential genetic susceptibility. Notably, sensitization has been reported in 1%-15% of the general adult population, with most data coming from the US and Europe (54, 93, 139–144). Although most studies focus on adults, AGS has also been documented in children (145, 146). Importantly, the presence of α-Gal-specific IgE does not necessarily equate to AGS. Most individuals with α-Gal-specific IgE are asymptomatic. Low levels of α-Gal IgE may go unnoticed until subsequent tick bites increase levels and trigger symptomatic reactions upon red meat consumption. Some studies have proposed diagnostic cut-off values by using α-Gal IgE levels and ratios to total IgE that are likely to result in clinically significant AGS. Mabelane et al. reported sIgE > 5.5 kU/L and a ratio > 2.12% for a 95% probability of AGS (147). Another study suggested sIgE ≥ 0.54 kU/L as the optimal cutoff point for assessing the diagnostic value of AGS particularly in allergy patients (148), whereas studies on a COVID-19 vaccine cohort and an AGS cohort found a cutoff of ≥ 1.4-1.5 IU/mL with a ratio of 3.4% (149, 150).

Risk exposure appears tightly linked to land-use patterns and environmental context. Individuals living in proximity to forested areas or within fragmented landscapes characterized by low-density or mixed-forest development exhibit higher risk of sensitization, suggesting that habitat edges where humans and ticks intersect are critical zones for exposure (151, 152). This association is reflected in several regional surveys. In Italy, for instance, sensitization was detected in 24.7% of rural residents but only in 1.2% of those living in urban settings (153). Comparable gradients were observed in Spain, where sensitization rates declined progressively from rural (37.7%) to semi-urban (15.4%) and urban (7.8%) populations (154). In less developed regions such as Ecuador, Zimbabwe and Kenya, elevated α-Gal-specific IgE levels were also recorded among rural populations; however, in these contexts, the antibodies are more likely to arise from parasitic infections, rather than from tick exposure (50, 155, 156). These geographic and environmental patterns mirror the distribution of tick species capable of transmitting α-Gal-bearing molecules. For instance, in a US military cohort, α-Gal sensitization clustered in the southeastern states and spatially overlapped with the known range of *A. americanum* (144). The majority of US AGS cases originate from regions where this tick is endemic (157). Moreover, in Spain, α-Gal IgE positivity was highest in northern regions, followed by central and Mediterranean zones, correlating with local tick species ecology (154). Individuals whose workplaces put them in frequent contact with wooded or grass-landed habitats, such as in agriculture or forest-related industries, hunters or wildlife practitioners may have an increased risk encountering ticks. Indeed, these activities were associated with a higher sensitization prevalence of 15% -58% (93, 139, 158–160). Together, these findings underscore that the global distribution of AGS is shaped by the ecological niches of specific tick species, and patterns of human settlement and land use. Since the first description of the syndrome in 2009, reported cases have however risen rapidly, and incidences are also likely to increase due to shifts in the expansion of tick habitats. As ectothermic organisms, ticks are highly sensitive to temperature and humidity. Particularly, with climate change, all tick-borne diseases, including AGS, are likely to expand to the north, as they are closely associated with the effect of warming on tick populations, vegetation and their hosts (161). Indeed, modeling studies already predict that ticks will shift northwards and to higher altitudes and become active for longer seasons under milder winters (162, 163).

Beyond demographic and environmental factors, biological determinants such as genetic susceptibility likely modify risk of sensitization. Among these, the ABO blood group system represents the most thoroughly investigated genetic factor. The B-group antigen differs from the α-Gal epitope by only a single fucose at the penultimate galactose residue, creating structural similarity that likely confers partial immunological tolerance through cross-reactivity. Consequently, individuals with blood group B or AB appear less susceptible to α-Gal sensitization and development of AGS. Multiple studies have demonstrated lower α-Gal-specific IgE titers and reduced disease prevalence within these groups (54, 92, 94, 164). Correspondingly, the frequency of blood group B is comparatively lower in Europe, the US and Australia, regions where AGS is increasingly reported, than in Africa or Asia, where α-Gal sensitization remains uncommon (42, 165). Nevertheless, exceptions exist, as individuals with blood group B/AB can still develop both α-Gal-specific IgE and AGS (145, 165).

## 6 Knowledge gaps and future directions

Despite substantial advances in the clinical characterization of α-Gal syndrome, key mechanistic questions remain unresolved regarding how tick exposure leads to durable IgE sensitization against α-Gal.

A central unresolved issue is why IgE responses to α-Gal arise only after tick exposure, whereas lifelong oral and microbial exposure to the same carbohydrate epitope overwhelmingly promotes immunological tolerance. This dichotomy strongly implicates the cutaneous route of exposure and the epithelial barrier as critical determinants of sensitization. Disruption of epithelial integrity is increasingly recognized as a driver of allergic disease, exemplified by the association between atopic dermatitis and peanut allergy, where skin barrier dysfunction facilitates transcutaneous sensitization (166, 167). Preclinical models further support this paradigm, demonstrating that cutaneous exposure to food allergens can induce IgE-mediated hypersensitivity (167). Within this framework, tick bites provide a unique and physiologically relevant model to study how immune responses at a damaged skin barrier can deviate from protective inflammation toward Th2-skewed immunity and IgE production. Notably, unlike most insect sting allergies, which predominantly induce IgE responses to protein antigens, tick bites are remarkably effective at promoting IgE responses to a carbohydrate epitope, raising the additional question of why tick bites favor glycan-directed sensitization.

Equally unclear are the initial immunological events after a tick bite that determine whether the host mounts a transient, protective inflammatory response or progresses toward a maladaptive Th2 response with sustained α-Gal-specific IgE production. Only a subset of tick-exposed individuals develops α-Gal sensitization, highlighting the importance of host-specific susceptibility factors. It is conceivable that the trajectory toward allergic sensitization is already imprinted in the early peripheral immune response following tick attachment and that protective anti-tick immunity can be distinguished from pro-allergic inflammation by discrete immune signatures shortly after exposure. Addressing this hypothesis, our group is conducting a clinical investigation (ClinicalTrials.gov ID: NCT07177729) to characterize early systemic immune responses after tick bites, with longitudinal follow-up of α-Gal–IgE–positive individuals and matched controls. This study aims to define immunological pathways linking skin injury to sensitization and to identify biomarkers predictive of disease development (168).

Beyond conventional adaptive immunity, the role of unconventional T cell subsets in AGS pathogenesis remains largely unexplored. CD1d-restricted iNKT cells recognize glycolipid antigens and are potent regulators of immune polarization. Increased frequencies of iNKT cells have been reported among peripheral blood mononuclear cells from AGS patients, and activated circulating CD1d-restricted NKT cells are detectable in α-Gal–allergic individuals (98, 100). These observations support the hypothesis that α-Gal–containing glycolipids contribute to AGS through CD1d-mediated activation of unconventional T cells and downstream Th2 programming. Ongoing work by Dr. Iweala and colleagues is directly addressing the role of glycolipids and unconventional T cell subsets in AGS pathogenesis (NIH project number: 5R01AI172112-03) (131, 169). In parallel, Dr. Erickson’s group is investigating whether iNKT cells and CCR6^+^ switched memory B cells contribute to atherosclerosis development in the context of α-Gal–specific IgE sensitization (NIH project number: 5R01AI172112-04) (169).

At the level of humoral immunity, major gaps remain regarding the B cell pathways that give rise to α-Gal–specific IgE. It is currently unclear whether IgE arises through direct class switching of naïve B cells or via sequential switching from pre-existing IgM- or IgG-expressing B cell intermediates. Addressing this fundamental question, ongoing work by the group of Prof. Biedermann aims to dissect α-Gal–specific B cell differentiation pathways and to define the contribution of distinct B cell subsets to pathogenic IgE formation (DFG project number: 527318848) (170). Complementary approaches focus on the immunoglobulin repertoire itself: analysis of variable gene sequences of α-Gal–specific IgE, currently pursued by the group of Dr. Smith (NIH project number: 1R01AI189950-01), is expected to clarify the cellular origin of pathogenic IgE and may ultimately enable prediction of individual susceptibility to α-Gal sensitization (169). Interestingly, α-Gal–specific IgE levels often decline over time in the absence of further tick exposure, suggesting that the IgE response may be maintained predominantly by plasmablasts or short-lived plasma cells rather than long-lived humoral memory (171, 172). Consistent with this immunological waning, case reports and clinical experience indicate that patients with AGS who avoid further tick bites and show declining α-Gal-specific IgE levels may be able to reintroduce mammalian meat into their diet (171, 173–175). Suggested thresholds for considering reintroduction include α-Gal-specific IgE levels below 0.35 kU/L, or even 0.1 kU/L (171, 175). Nevertheless, these recommendations are based on observational data rather than systematic studies defining standardized criteria for safe dietary reintroduction across patient populations. As such, current practice relies on individualized risk assessment, declining α-Gal-specific IgE levels and specialist supervision.

At present, management of AGS relies almost exclusively on avoidance of mammalian meat and prevention of further tick bites. This strategy is particularly challenging due to incomplete labeling of mammalian-derived products and the widespread presence of α-Gal in gelatin-containing foods, pharmaceuticals, and medical products. Consequently, several therapeutic approaches are under investigation. Oral immunotherapy (OIT) with red meat has shown promising results in adult patients and a pediatric case, inducing tolerance in selected individuals (176–179); however, renewed tick exposure has been associated with recurrence of anaphylaxis, underscoring the fragility of this approach (177). Alternative strategies include nanoparticle-based immunotherapy: α-Gal–encapsulating biodegradable nanoparticles have demonstrated both prophylactic and therapeutic efficacy in murine models, reducing Th2 cytokine production, allergen-specific IgE levels, and effector cell activation (180). Related approaches using poly-L-lysine–based α-Gal glycoconjugates aim to selectively inhibit α-Gal–specific IgE production, representing another promising avenue for immune modulation (181).

Finally, growing insight into the immunomodulatory networks induced by tick feeding has catalyzed interest in tick-targeted vaccines as a preventive strategy. In line with this, two human studies aim to establish whether repeated, controlled exposure to *I. scapularis* can induce measurable anti-tick immunity in humans. Both studies monitor innate and adaptive responses via skin biopsies and blood sampling to develop a model of acquired tick resistance in humans. These efforts would provide an important translational bridge toward the development of effective anti-tick vaccines (ClinicalTrials.gov ID: NCT05036707 and NCT05965635) (182, 183). An mRNA-based vaccine encoding 19 *I. scapularis* salivary proteins has recently been shown to impair tick feeding and prevent *Borrelia burgdorferi* transmission in guinea pigs, highlighting the feasibility of inducing acquired tick resistance (184). Extending this concept, induction of anti-tick immunity may represent a novel strategy to prevent α-Gal sensitization altogether. Accordingly, mRNA-based vaccines targeting *A. americanum* and other AGS-relevant tick species are currently under investigation by the group of Dr. Narasimhan (NIH project number: 1R21AI186078-01A1).

## 7 Conclusion

As the prevalence of AGS continues to rise globally, sustained investigation into its epidemiology and underlying mechanisms is essential to improve both management and prevention strategies. Recent advances have begun to elucidate key molecular and immunological determinants of sensitization, drawing on insights from meat-allergic patients, sensitized but asymptomatic individuals, tick-exposed cohorts and preclinical models. Despite this progress, substantial gaps remain in our understanding of AGS pathogenesis, underscoring the need for refined experimental models and innovative methodological approaches. Central to AGS is the tick bite, which delivers a complex and immunomodulatory mixture of salivary components into the skin, thereby creating a unique context for immune activation. The ensuing IgE response to the carbohydrate epitope α-Gal challenges traditional concepts of glycan immunogenicity, as it clearly involves T cell help. These T cells exhibit a pronounced Th2 bias and are likely critical for driving class-switch recombination to IgE from pre-existing IgM- or IgG-committed B cells. Clinically, avoidance of mammalian meat and related products remains the basis of symptom management, while prevention of further tick exposure is currently the only effective strategy to reduce or eliminate α-Gal–specific IgE. Looking ahead, ongoing and future studies promise to resolve outstanding questions regarding immune deviation, ultimately enabling more precise risk stratification and the development of targeted preventive and therapeutic interventions for AGS.
